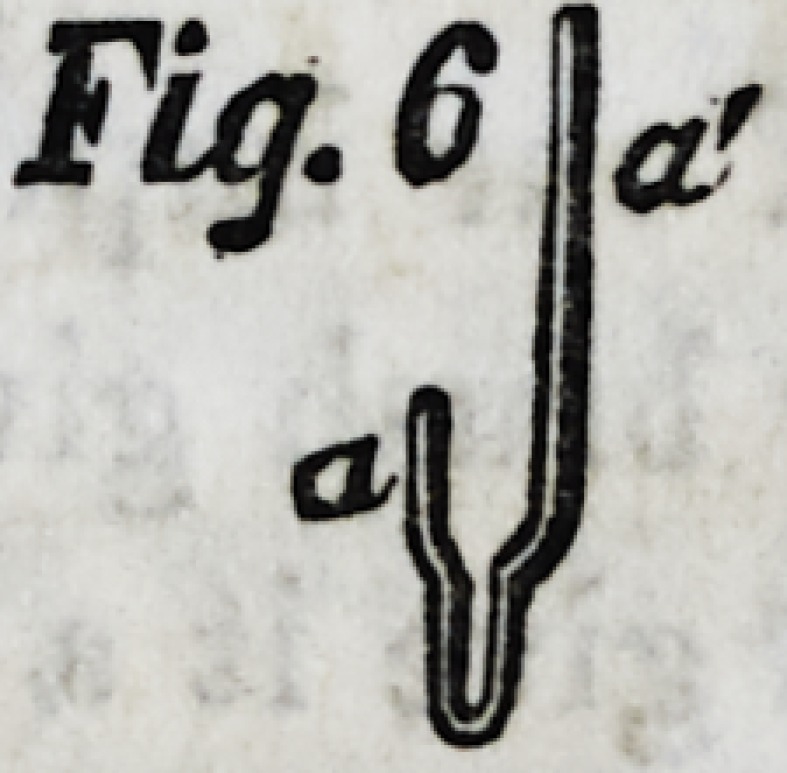# Hayes' Method of Mounting Artificial Teeth

**Published:** 1857-04

**Authors:** 


### ARTICLE XIV.

### *Hayes' Method of Mounting Artificial Teeth.*

CONSPICUOUS among the many quite recent improvements in dentistry stands the construction of continuous or solid gums, for connecting the teeth with each other and with the plate, when a full set or any considerable portion of a set is supplied. Although the validity of the patent therefor has been, and still is, sharply contested, we believe the material manufactured by Dr. John Allen, of this city, composed of flinty substances which melt at a little less heat than the teeth, is the most popular for the purpose, as it is almost free from any disposition to contract, and thus to warp the plate when exposed to the intense heat required in the baking process. The old process still in vogue with many dentists, employs teeth having each a corresponding short portion of gum cast on it, ready for attaching to the plate by simple riveting, but, although it requires much greater mechanical skill in the operator, the really progressive men in the profession are now adopting the continuous gum, on account, partly, of its greater strength and superior appearance, but mainly on account of its cleanliness. The patch up sets, made of teeth and gums in fragments simply riveted, are full of joints, forming cavities where food and saliva lodge and become offensive unless cleansedwith extreme care, and it is obviously impossible, from its construction, ever fully to cleanse the narrow and crooked fissures thus made.

The improvement represented in the accompanying engravings, relate to methods of attaching the teeth to the plates by wires, etc., which are soldered before the gum composition is laid and finally covered by the same. The earthy composition of the gum is strong, but not sufficent of itself to hold the teeth with certainty in biting very hard substances, and even if it were, a connection of some kind is always absolutely necessary to confine the teeth in exactly the right positions until the composition hardens. We cannot be expected to teach the profession all the details for applying this invention, nor all the points of difference between this and other methods, but will endeavor to set forth its general features.

The heat necessary to consolidate properly the porcelain or earthen gums, forbids the employment of the usual metals in connection. Gold or silver, which melt at from 1800° to 2300° Fah., would be of no service as bands or ties, and even when used as solder for the quite unfusible platinum, melt, and would, if used in any sensible quantities, flow away unless confined by the surrounding earths. In this invention, platinum plates are used as a foundation, and platinum wires as the means of attaching the teeth thereto, after which the whole is nicely covered with the melted composition, taking care to fill all the interstices between the wires, and to apply the proper oxyds of gold, etc., for producing the proper pink tint natural to the real healthy gum, after which the whole is melted at a very high heat and turned out perfect.

Fig. 1, is a set of teeth represented partly supplied with the gum composition. Fig. 2 is a side view of the set before the composition was applied; Fig. 3, is a vertical section through the same, the section passing through the center of a tooth; Fig. 4, is a similar section betwen two teeth; Fig. 5, is a tooth properly wired according to this invention before its introduction into the set, and Fig. 6 is the wire (a flattened strip of platina) introduced in the tooth before it is baked. We may remark here, that these teeth, as well also as those above mentioned more generally employed, are manufactured on a large scale from a kind of porcelain, and sold to the profession, and are not, as supposed by many, made up on the spot where used, by the skill of the operating dentist alone.

Commencing with Fig. 6, and proceeding backwards, we may describe *a a*, as the short bent wire introduced deeply in the base of each tooth in the course of manufacture. Fig. 5, is a tooth complete with the ends of the wire projecting. Fig. 4, shows a tooth in place, B, being a plate accurately swaged to correspond with the form of the gums and roof of the mouth, and *e*, a smaller plate similarly swaged to cover the roof of the mouth alone. C, is the earthenware material. The little circle, *c*, shows a cross section of a stout wire which travels continuously around the whole set to steady them, and *f*, is a brace stretching from *c* to D, and soldered to each. Fig. 3, shows similar parts, but with the short end, *a*, of the original tooth wire bent around and soldered to *c*, while the long end, *a′*, is extended up and soldered to B. Fig. 2, shows all the parts in place, and indicates, by the letter *d*, a kind of folded edge (equally visible on Figs. 3 and 4) formed on the edge of B. Fig. 1, explains itself, and it is only necessary to add that the additional plate, *e*, is soldered on, and the edge, *d*, is turned down, both for the same purpose, *i. e.*, to offer better facilities for joining the gum composition, earthenware, or porcelain, C, to the other parts by a perfectly smooth and flush joint, so that the set, when complete, shall be as nearly like the natural mouth as possible. Teeth thus set are much preferable to the old method on every account, and we believe usually cost considerably more.—*Sci. Am.*

## Figures and Tables

**Fig 1 f1:**
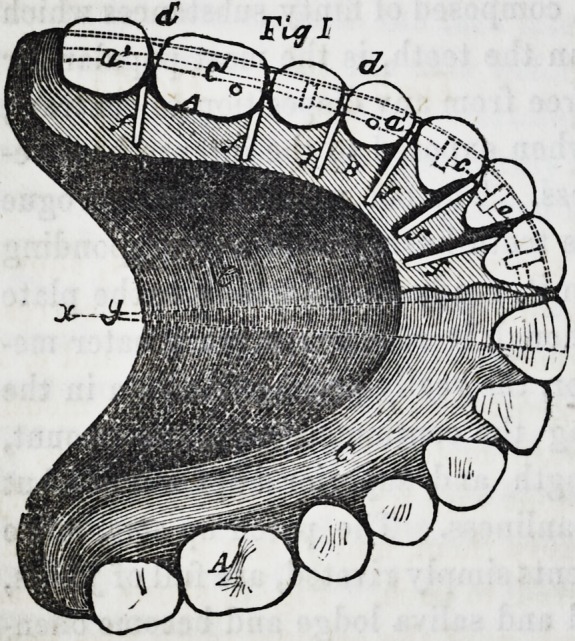


**Fig. 2 f2:**
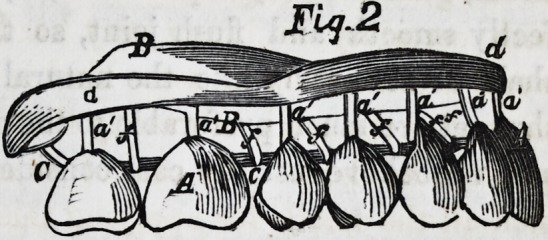


**Figure f3:**
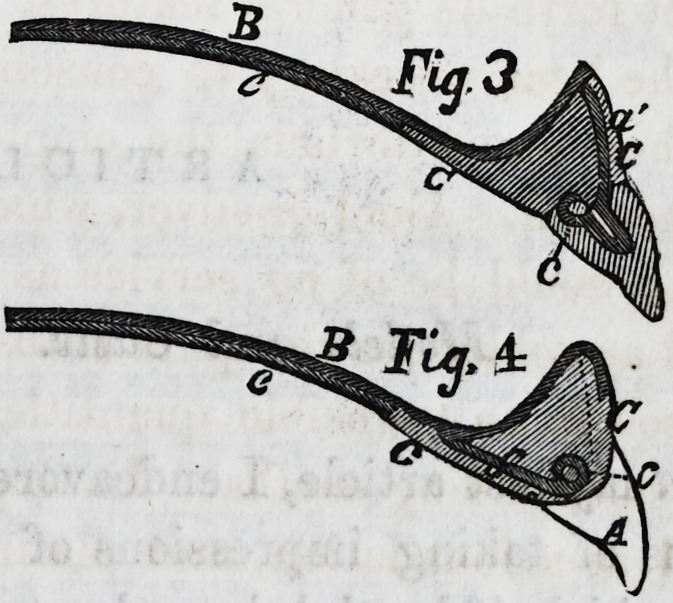


**Fig. 5 f4:**
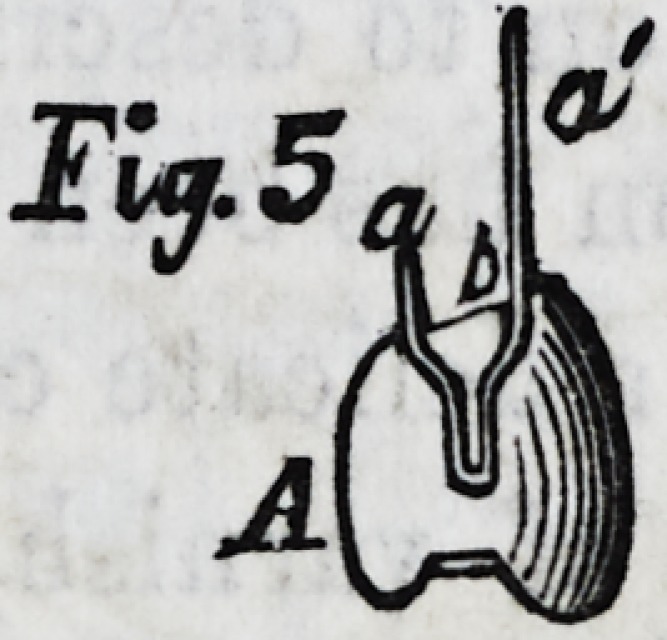


**Fig. 6 f5:**